# *In vitro* and *in vivo* anthelmintic efficacy of peppermint (*Mentha x piperita* L.) essential oil against gastrointestinal nematodes of sheep

**DOI:** 10.3389/fvets.2023.1232570

**Published:** 2023-08-10

**Authors:** Filip Štrbac, Slobodan Krnjajić, Dragica Stojanović, Radomir Ratajac, Nataša Simin, Dejan Orčić, Laura Rinaldi, Elena Ciccone, Maria Paola Maurelli, Giuseppe Cringoli, Antonio Bosco

**Affiliations:** ^1^Institute for Multidisciplinary Research, University of Belgrade, Belgrade, Serbia; ^2^Department of Veterinary Medicine, Faculty of Agriculture, University of Novi Sad, Novi Sad, Serbia; ^3^Scientific Veterinary Institute Novi Sad, Novi Sad, Serbia; ^4^Department of Chemistry, Biochemistry and Environmental Protection, Faculty of Sciences, University of Novi Sad, Novi Sad, Serbia; ^5^Department of Veterinary Medicine and Animal Production, University of Naples Federico II, CREMOPAR, Naples, Italy

**Keywords:** *Mentha x piperita*, essential oil, gastrointestinal nematodes, sheep, anthelmintic efficacy

## Abstract

Nowadays, the exclusive use of commercial anthelmintics for the treatment of gastrointestinal nematode infections in ruminants is less sustainable due to anthelmintic resistance, as well as the problem of drug residues in animal products and the environment. Therefore, an integrated therapeutic approach is needed, including the search for alternatives to synthetic anthelmintic drugs. The aim of this study was to evaluate the possibility of using the essential oil of peppermint (*Mentha x piperita* L.) in the control of gastrointestinal nematodes in sheep. For this purpose, the *in vitro* and *in vivo* anthelmintic efficacy of this oil and the toxic effects on the hosts were examined. In the *in vitro* egg hatch test, ovicidal activity varied from 21.0–90.3% depending on the concentration of essential oil used (0.0125, 0.025, 0.049, 0.195, 0.781, 3.125, 12.5, and 50 mg/mL). To some extent, anthelmintic efficacy was confirmed in the *in vivo* fecal egg count reduction test at a mean dose of 150 mg/kg, with an average reduction of nematode eggs of 26.9 and 46.0% at Days 7 and 14 after treatment, respectively. Furthermore, no toxic effects of applied oil were observed on sheep behavior, kidney, or liver function. The main compounds identified by gas chromatography–mass spectrometry analyzes were menthol (32.6%), menthone (22.0%), menthyl-acetate (10.0%), and isomenthone (9.39%). Due to their complex chemical compositions, numerous bioactive ingredients, and natural origin, herbal formulations represent a potentially valuable alternative for the control of gastrointestinal nematodes in sheep. In this context, the results of the present study showed that peppermint essential oil is one of the promising candidates. Further studies should be performed to collect more data on the safety profile of *M. piperita* EO in treated animals to find the most appropriate formulation for use in field conditions and to test it against resistant gastrointestinal nematode populations.

## 1. Introduction

Gastrointestinal nematodes (GINs) are one of the most important endoparasites in grazing animals, especially ruminants (1). In sheep, these parasites may cause various negative effects including subclinical disease with weight loss and reduced animal production, or clinically manifested disease with signs of anemia, diarrhea, protein loss, anorexia, decreased immunity, and fertility (2–4). In the case of a high worm burden, a possibly fatal outcome may occur (5). Therefore, due to decreased productivity, high treatment costs, and possible death of the animals, GIN parasitism can have a huge economic impact (6). Commercially available anthelmintics have been used successfully for decades to control these parasites (7). However, as a result of their irrational use, anthelmintic resistance (AR) has developed, whereby the following are considered high-risk factors: underdosing, overfrequent treatments, mass treatment, and single-drug regimens (8, 9).

Thus, increasing resistance of nematodes to the well-known group of anthelmintics such as benzimidazoles, macrocyclic lactones, and imidazothiazoles has been reported, sometimes simultaneously to several different classes (10, 11). The problem is also present in novel anthelmintic groups such as monepantel, an amino-acetonitrile derivative (12). Annual economic losses resulting from the development of AR are estimated at €38 million in Europe, with expected growth in the future (10). Other problems associated with the use of commercial anthelmintics are residues present in animal products such as meat and milk or in the ecosystem and biodiversity. This represents a serious problem associated with many currently available chemotherapeutic drugs, along with the increasing price (13, 14). All the above-mentioned issues suggest the urgent need for developing novel strategies for future treatments (4).

As a possible solution for achieving sustainable control of sheep GINs, an integrated approach has been proposed by many researchers (1, 15–19). This refers to the rational use of commercial anthelmintics, along with the use of alternatives. On the one hand, the rational use of anthelmintics should be based on refugia strategies such as targeted treatments (TT) or targeted selective treatments (TST), which imply only the treatment of herds or animals that require it due to clinical or economic reasons (19–21). Besides, the rational use of commercial drugs may refer to the combination or rotation of anthelmintics from different chemical classes in order to avoid the excessive use of only one drug (5, 7). On the other hand, integrated control implies the incorporation of various alternative strategies into the practice, such as genetic selection of sheep naturally resistant to GINs, pasture management, dietary manipulation, vaccine development, biological control (direct–use of nematophagous fungi, bacteria, and predatory nematodes or indirect–use of earthworms or dung beetles), or the use of plant-based anthelmintics (condensed tannins, various extracts, or essential oils) (6, 15, 19, 22, 23).

As aromatic, complex, and concentrated mixtures of volatile, nonpolar compounds, essential oils (EOs) belong to the group of plant secondary metabolites that are responsible for enabling plants to be competitive in their own environment (24, 25). Thus, these mixtures contain various compounds such as terpenes, terpenoids, and phenylpropanoids that can be used for various pharmacological purposes (26, 27). EOs and their compounds can be obtained from plants by various extraction methods, and along with their extracts, they are increasingly being used in veterinary medicine in recent times. Among the others, indications for their application include the use as coccidiostats to boost immunity and improve the performance of poultry, to prevent diarrhea in piglets (due to their antimicrobial properties), as fish diet supplementation, to use against *Varroa destructor* in beekeeping or against *Malassezia pachydermatis* and ticks in pets, etc. (28–31). In ruminants, the possible benefits of EOs among the others include the improvement of ruminal fermentation and digestion and the reduction of methanogenesis and nitrogen excretion (29). In addition, various studies have demonstrated the efficacy of EOs against GINs in sheep. The list is wide and includes oregano, thyme, coriander, tea tree, and lavender as well as various species of lemongrass, eucalyptus, lippia, mint, etc. (32, 33).

Peppermint (*Mentha x piperita* L.) represents a well-known aromatic and medicinal herb derived from crossing between spearmint (*Mentha spicata* L.) and water mint (*Mentha aquatica* L.). It belongs to the Lamiaceae family and is native to the Mediterranean region but is now cultivated in various parts of the world (34). Peppermint is one of the most important plant species in the pharmaceutical and cosmetic industries, whose EO is widely produced and used. Many pharmacological properties of peppermint have been previously demonstrated and include analgetic, antiviral, antibacterial, antifungal, and antiparasitic effects (35, 36). Moreover, the effect of *M. piperita* EO against sheep GINs was also demonstrated in various studies, whereby it showed promising results against different parasite stages (37–39). However, only the efficacy under laboratory conditions has been proven so far, which requires the confirmation of these results in field trials. Therefore, along with *in vitro*, the aim of the present study was to examine the *in vivo* effect of *M. piperita* EO against GINs of sheep, as well as to evaluate the safety of its use in the hosts.

## 2. Materials and methods

### 2.1. Chemical analyzes

*Mentha piperita* EO was obtained from the Institute of Field and Vegetable Crops, Novi Sad, Serbia, whereby the chemical composition (qualitative and semiquantitative characterization) of the tested EO was determined by gas chromatography–mass spectrometry (GC–MS) at the Department of Chemistry, Biochemistry, and Environmental Protection, Faculty of Sciences, University of Novi Sad, Serbia. The exact parameters regarding the technical conditions for the analysis are described in Knežević et al. (40) and Štrbac et al. (41) as follows: injection volume of EO 1 μL; injector temperature 250°C; split ratio 1:10; carrier gas helium; flow rate: 1 mL/min; capillary column: HP-5 (30 m × 0.25 mm, 0.25 μm); temperature program 50–270°C; ion source temperature 230°C; electron energy 70 eV; quadrupole temperature 150°C. The compounds were identified by comparison of mass spectra with data libraries (Wiley Registry of Mass Spectral Data, 7th ed., and NIST/EPA/NIH Mass Spectral Library 05) and confirmed by comparison with arithmetic retention indices (AI) with literature data (42). Diesel oil, containing C8– C28 n-alkanes, was used as a standard for the determination of retention indices. The relative amounts of the components, expressed in percentages, were calculated by the normalization procedure according to the peak area in the total ion chromatogram.

### 2.2. *In vitro* test–egg hatch test

The remaining analyzes were performed at the Regional Center for Monitoring of Parasitosis (CREMOPAR) located in Eboli (SA), Italy, in 2021. For the *in vitro* examination of the anthelmintic potential of *M. piperita* EO, the egg hatch test (EHT) was chosen. GIN eggs were collected from fecal samples taken directly from the rectal ampulla of sheep (*n* = 40) with natural-mixed infection, and processed within 2 h of collection by the recovery method (43). To separate the eggs from the feces, samples were first pooled, homogenized, and filtered under running water through meshes of different sizes (1 mm, 250 μm, 212 μm, and 38 μm). GIN eggs that were retained on the smallest mesh were washed with distilled water and centrifuged at 1500 rpm for 3 min, after which the supernatant was discarded. Then, centrifugation was performed using a 40% sugar solution to float the eggs, which were then isolated into new tubes and mixed with distilled water. Finally, a few more centrifugations were performed to remove the pellets and obtain an aqueous solution containing GIN eggs.

Similar to our previous study (41), EHT was performed at eight different concentrations (50, 12.5, 3.125, 0.781, 0.195, 0.049, 0.025, and 0.0125 mg/mL) of the tested EO obtained by the dilution. For this purpose, 24-well plates were used in which each concentration of EO was emulsified with 3% Tween 80. An aqueous solution (40 mL) containing approximately 150 GIN eggs was added to each well, which was then completed with distilled water to reach a final volume of 0.5 mL in the wells. The positive control was thiabendazole at the two lowest concentrations used for EO, and the negative controls were 3% Tween 80 at the amount used for the emulsion, as well as distilled water. After an incubation period of 48 h at 27°C, the GIN eggs and the hatched first-stage (L_1_) larvae were counted under an inverted microscope, and the inhibition of hatchability was calculated. The experiment was performed in three replicates, and the obtained values were expressed as arithmetic mean ± standard deviation.

### 2.3. *In vivo* test–fecal egg count reduction test

The *in vivo* trial was conducted in two different farms in southern Italy (Campania region), where the prevalence of GINs is high (3). In Farm 1, the animals were free-range, and in Farm 2, the animals were kept in boxes during treatment. Sheep with natural-mixed infection and different worm burdens were used, mainly the Lacaune/Bagnolese mixed dairy breed, which was homogeneous in terms of age (2 years ±0.5) and grazing season, with an average body weight (b.w.) of 50 kg ± 5. The tested animals were fed with pasture and forage (barley and maize grains), and the diet was not changed during the experiment. None of the commercial treatments were applied at least 6 months before the trial, and the animals fasted and remained fasting until 2 h after administration of EO. In both farms studied, sheep were divided into three different treatment groups:

G1: 150 mg/kg of *M. piperita* EO (*n* = 12 animals/group/farm).

G2: 3.8 mg/kg of albendazole, positive control (*n* = 12 animals/group/farm).

G3: 50 mL of sunflower oil per animal, negative control (*n* = 12 animals/group/farm).

The EO formulation was prepared by diluting *M. piperita* EO in sunflower oil (1,4.5) to avoid the effect of pure oil on the mucous membranes of the gastrointestinal tract. In an attempt to ensure the largest possible number of active ingredients from EO reaching the target sites (abomasum and intestine), the formulation (as well as the controls) was applied directly into the rumen of the animals. Individual fecal samples were collected rectally before treatment (D0) and 7 and 14 days after treatment (D7 and D14) and stored at 4°C before processing. Collected samples were analyzed using the novel Mini-FLOTAC technique (44) with a detection limit of five eggs per gram (EPG) of feces, using a sodium chloride flotation solution (specific gravity = 1.200). Percentages of EPG reduction were evaluated at D7 and D14 by calculating means within each group, whereby the final results for each group were presented as arithmetic means of the two farms examined.

### 2.4. Coproculture

The coproculture study was conducted according to the protocol developed by the UK Ministry of Agriculture, Fisheries, and Food (45) in order to identify the GIN genera in the sampled farms and the changes in their population after treatment. Before storage at 4°C, an equal amount of feces was collected from each sample to form a pool for each group formed for the *in vivo* test (*M. piperita* EO, albendazole, and sunflower oil) at different time points (D0, D7, and D14). The developed third-stage larvae (L_3_) were identified based on the morphological keys proposed by van Wyk and Mayhew (46). The identification and percentage of each nematode genus was performed on 100 L_3_, whereby all larvae were identified if a sample contained 100 or fewer L_3_. In this way, the percentage of each genus could be determined from the total number of larvae identified (41).

### 2.5. Toxicity studies

All treated animals were observed clinically for the presence of potential adverse effects, with particular attention to their feeding, defecation, and behavior. In addition, blood samples were collected from the jugular vein at D0 and D14 to evaluate the effects of the used EO on blood parameters. For hematological parameters, blood samples were collected in vacuum tubes containing EDTA and processed shortly thereafter (within 2–4 h), whereas, for the biochemical parameters, blood was collected in vacuum tubes without coagulant and subsequently analyzed. For the latter, special attention was paid to urea, creatinine, and AST and GGT, which represent parameters of renal and hepatic function, respectively.

### 2.6. Statistical analyzes

Inhibition of Hatchability (IH) in the EHT was calculated using the following formula proposed by Coles et al. (47) and Pinto et al. (22):

IH (%) = number of eggs/(number of eggs + number of L1 larvae) × 100.

For the mutual comparison of the ovicidal effect of different concentrations as well as with controls, one-way analysis of variance (ANOVA) with *post hoc* Tukey’s test was performed with a value of p threshold of 0.05. Nonlinear regression/logarithmic distribution was used to assess the presence of a dose-dependent effect and calculate the half-maximal inhibitory concentration (IC_50_) (48).

On the FECRT, the reduction in mean EPG in each treatment group at each time point was calculated using the following formula (48, 49):

FECR (%) = (1 − (T_2_/T_1_ × C_1_/C_2_)) × 100.

In this formula, T_2_ represents the EPG after treatment (D7 or D14); T_1_ represents the EPG before treatment (D0), C_1_ represents the EPG before treatment in the negative control group and C_2_ represents the EPG after treatment (D7 or D14) in the negative control group. The values obtained were compared using the two-way ANOVA followed by Tukey’s test in order to evaluate the differences in effect within a group and location for different days, as well as within the same day and location for different groups (value of p threshold of 0.05).

Finally, for the analysis of the results of the hematological and biochemical blood analyzes, a two-way ANOVA was also performed. For the comparison of values in the same group at D0 and D14 after treatment, the *post hoc* Sidak’s test (*p* < 0.05) was used, while for the comparison of values obtained for different groups on the same day, *post hoc* Tukey’s test (*p* < 0.05) was used.

## 3. Results

### 3.1. Chemical analyzes

**Graph 1:**
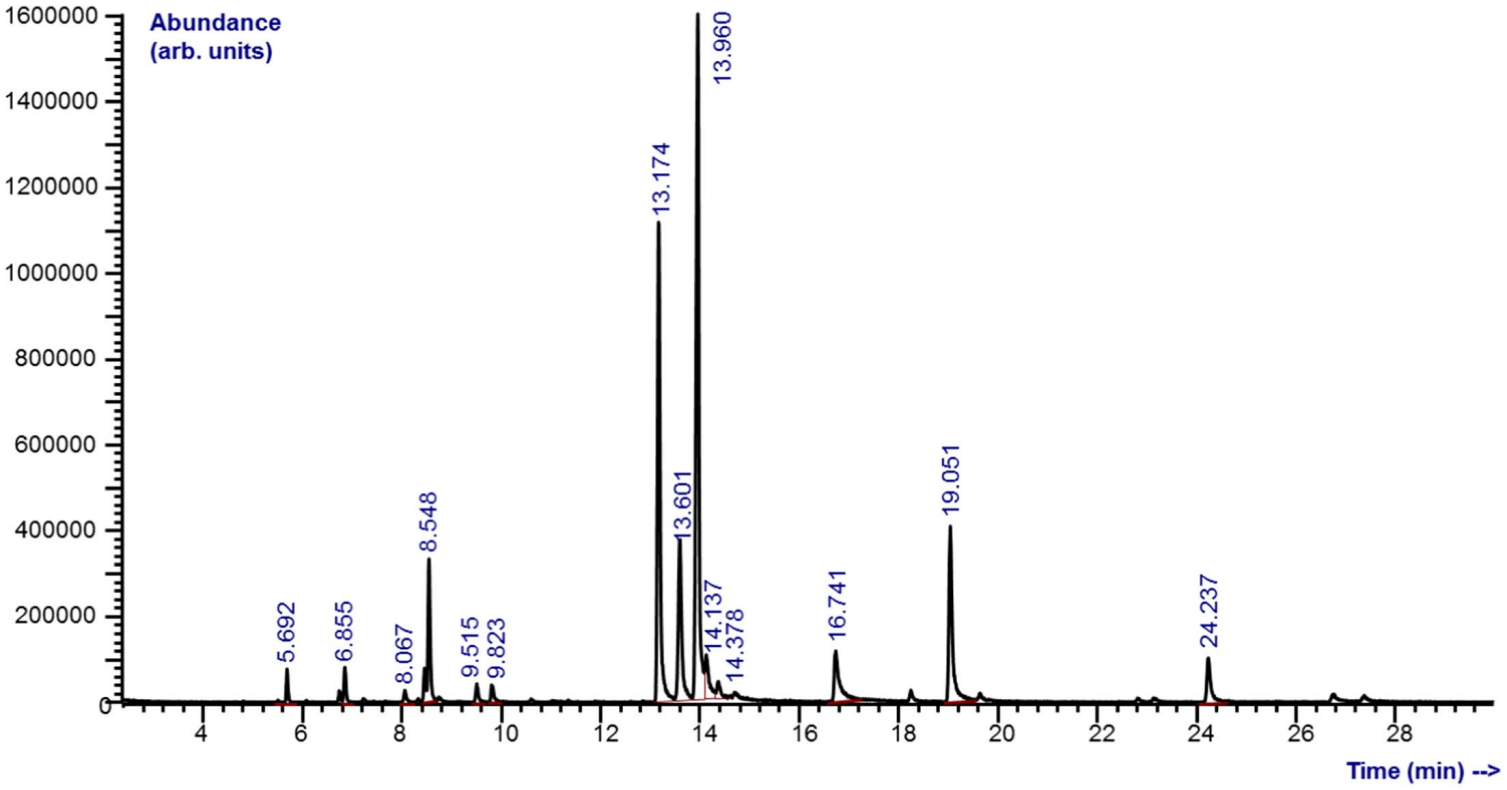
*Mentha x piperita* L. essential oil by gas chromatography–mass spectrometry analyzes.

Chemical analyzes revealed a complex composition of *M. piperita* EO with a total of 31 different compounds, of which 27 were identified (Graph 1; Table 1). The compounds belong to different chemical classes, where monoterpenes - alcohol (menthol, 32.6%), ketones (menthone, 22.0%; isomenthone, 9.39%), ester (menthyl acetate, 10.0%), and ether (eucalyptol, 5.22%) were predominant (Graph 1).

**Table 1:** Chemical composition (% of total peak area) of the *Mentha x piperita* L. essential oil determined by gas chromatography–mass spectrometry analyzes.

AI*	Compound	% of total peak area
925	α-Thujene	0.05
932	α-Pinene	0.90
946	Camphene	0.03
971	Sabinene	0.36
976	β-Pinene	1.15
990	Myrcene	0.08
1,016	α-Terpinene	0.46
1,023	p-Cymene	0.11
1,027	Limonene	1.17
1,029	1,8-Cineole	5.22
1,035	cis-Ocimene	0.19
1,057	γ-Terpinene	0.67
1,065	n.i.	0.65
1,087	Terpinolene	0.12
1,100	trans-Sabinene hydrate	0.05
1,107	n.i.	0.04
1,152	Menthone	22.0
1,163	Isomenthone	9.39
1,172	Menthol	32.6
1,176	Terpinen-4-ol	3.26
1,182	iso-Menthol	1.29
1,190	α-Terpineol	0.46
1,238	Pulegone	4.45
1,274	neo-Menthyl acetate	0.49
1,293	Menthyl acetate	10.0
1,307	iso-Menthyl acetate	0.45
1,383	β-Bourbonene	0.18
1,391	n.i.	0.25
1,417	β-Caryophyllene	2.78
1,480	Germacrene D	0.73
1,495	n.i.	0.46
Total % of identified compounds		100.0

*AI–arithmetic retention index.

### 3.2. *In vitro* test–egg hatch test

**Graph 2:**
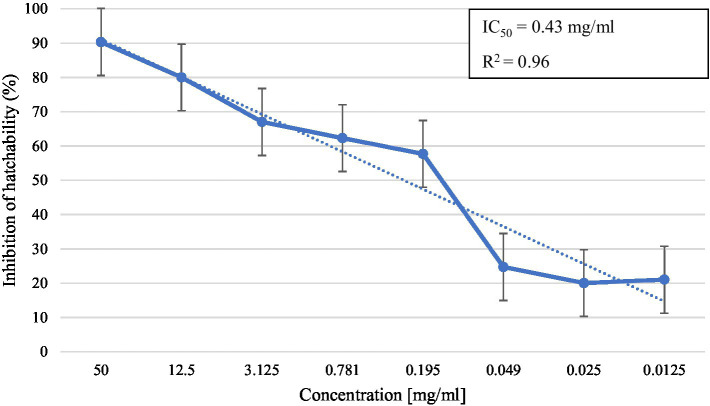
Inhibition of egg hatchability at different concentrations of *Mentha x piperita* L. essential oil.

In the *in vitro* test, *M. piperita* EO showed ovicidal activity with inhibition of hatchability ranging from 20.0 to 90.3% depending on the concentration used (Table 2). The highest concentration showed similar activity with thiabendazole at both concentrations tested (*p* > 0.05), whereas all tested EO concentrations showed significantly higher activity than negative controls (*p* < 0.05). The calculated *R*^2^ value was 0.96, suggesting a dose-dependent activity with a determined IC_50_ value of 0.43 mg/mL (Graph 2).

**Table 2:** Ovicidal activity of *Mentha x piperita* L. essential oil against gastrointestinal nematodes eggs (*in vitro*–egg hatch test).

Concentration (mg/mL)	Inhibition of hatchability (%)
50	90.3 ± 2.08^A^
12.5	80.0 ± 2.00^B^
3.125	67.0 ± 2.65^C^
0.781	62.3 ± 2.52^CD^
0.195	57.7 ± 1.53^D^
0.049	24.7 ± 2.52^E^
0.025	20.0 ± 1.00^EF^
0.0125	21.0 ± 1.00^E^
Control (+)^a^	96.3 ± 1.53^A^
Control (+)^b^	95.0 ± 1.00^A^
Control (−)^c^	14.2 ± 3.34^F^
Control (−)^d^	6.60 ± 1.92^G^

Uppercase compares means between different concentrations as well as controls. Different letters indicate significant differences (*p* < 0.05). Control (+)^a^ - thiabendazole, 0.025 mg/mL; control (+)^b^ - thiabendazole, 0.0125 mg/mL; control (−)^c^ - 3% Tween 80, v/v; control (−)^d^ - distilled water.

### 3.3. *In vivo* test*–*fecal egg count reduction test

**Graph 3:**
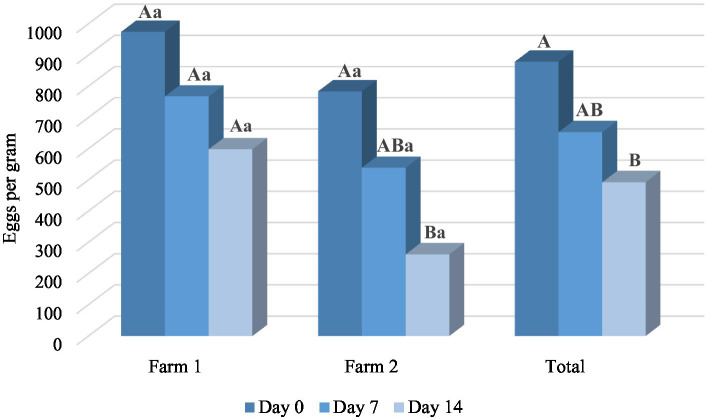
The mean number of eggs per gram at different time points in *Mentha x piperita* L. essential oil treatment groups. Uppercase compares means between different time points within one location and lowercase between two farms at the same time point. Different letters indicate significant differences (*p* < 0.05).

The results of *in vivo* trial also showed the anthelmintic potential of *M. piperita* EO with a mean efficacy of 26.86% (Day 7) and 46.04% (Day 14) (Table 3). Applied oil significantly reduced the number of EPG in comparison with a value obtained before the treatment (*p* < 0.05) in Farm 2, as well as in total. In general, the effect of EO was better on Farm 2 (Graph 3) and reached an efficacy of 33.63 and 63.35% on Days 7 and 14, respectively. However, EPG values in two different farms were not significantly different (*p* > 0.05) in the *M. piperita* group, in contrast to the control groups where EPGs were significantly higher in Farm 1 (*p* < 0.05).

**Table 3:** Eggs per gram (mean ± standard deviation) values and efficacy (%) of *Mentha x piperita* L. essential oil against gastrointestinal nematodes of sheep (*in vivo*–fecal egg count reduction test).

Treatment		*M. piperita* EO, 150 mg/kg		Albendazole, 3.8 mg/kg (control +)		Sunflower oil, 50 mL (control -)	
Location/Time point		EPG	Reduction	EPG	Reduction	EPG	Reduction
Farm 1	Day 0	974.6 ± 709.2^Aa^	/	983.3 ± 1160.1^Aa^	/	1279.6 ± 1092.3^Aa^	/
	Day 7	767.5 ± 637.9^Aa^	20.08%	17.9 ± 20.7^Bb^	98.16%	1260.9 ± 1052.6^Aa^	/
	Day 14	598.3 ± 707.5^Aa^	28.73%	66.7 ± 70.8^Ba^	92.17%	1102.3 ± 841.8^Ab^	/
Farm 2	Day 0	783.8 ± 770.0^Aa^	/	377.9 ± 311.0^Aa^	/	548.3 ± 551.5^Aa^	/
	Day 7	538.8 ± 505.6^Aa^	33.63%	0.88 ± 2.89^Bb^	99.79%	567.9 ± 517.0^Aa^	/
	Day 14	261.3 ± 295.8^Ba^	63.35%	7.08 ± 12.5^Bb^	97.94%	498.8 ± 518.8^Aa^	/
Total	Day 0	879.2 ± 739.6^Aa^	/	680.6 ± 735.6^Aa^	/	914.0 ± 821.9^Aa^	/
	Day 7	653.2 ± 572.0^Ba^	26.86%	9.39 ± 11.8^Bb^	98.98%	914.4 ± 784.8^Aa^	/
	Day 14	492.8 ± 501.7^Ba^	46.04%	36.9 ± 41.7^Cb^	95.06%	800.6 ± 680.3^Aa^	/

Uppercase compares means between different time points within one treatment group and location (Farm 1, Farm 2, or in total); lowercase compares means between different treatment groups at one time point and one location. Different letters indicate significant differences (*p* < 0.05). EO, essential oil; EPG, eggs per gram.

### 3.4. Toxicity studies

No side effects were noticed during clinical observation of the tested animals on Days 7 and 14 after the treatment. Similarly, values of hematological parameters did not change significantly (*p* > 0.05) after EO administration (Supplementary Table S1). In the biochemical blood analyzes, the values of urea and creatinine were also similar on Day 14 compared to Day 0, whereas liver enzymes showed a significantly lower activity after treatment with *M. piperita* EO and albendazole (Table 4).

**Table 4:** The effect of *Mentha x piperita* (L.) essential oil on serological parameters evaluating kidney and liver function in treated animals–in total from both examined farms.

Group	Day	Urea (mg/dl)	Creatinine (mg/dl)	AST (UI/l)	GGT (UI/l)
*Mentha piperita* (L.) EO	0.	29.3 ± 7.24^Aa^	12.9 ± 3.48^Aa^	225.2 ± 47.47^Aa^	69.8 ± 8.01^Aa^
	14.	30.3 ± 3.93^Aa^	13.5 ± 2.91^Aa^	156.8 ± 49.99^Ba^	65.0 ± 6.09^Ba^
Albendazol (control +)	0.	27.6 ± 9.45^Aa^	12.4 ± 2.75^Aa^	173.3 ± 82.72^Aa^	72.0 ± 8.86^Aa^
	14.	28.4 ± 6.07^Aa^	13.4 ± 3.68^Aa^	163.0 ± 50.72^Aa^	66.5 ± 5.54^Ba^
Sunflower oil (control –)	0.	29.8 ± 7.87^Aa^	12.4 ± 2.84^Aa^	189.7 ± 72.63^Aa^	72.5 ± 7.82^Aa^
	14.	29.3 ± 4.91^Aa^	12.8 ± 3.04^Aa^	206.9 ± 56.32^Aa^	73.7 ± 5.30^Aa^

Uppercase compares means between values at different time points within one treatment group and lowercase values at the same time points between different treatment groups. Different letters indicate significant differences (*p* < 0.05); AST, aspartate aminotransferase; GGT, gamma-glutamyltransferase; EO, essential oil.

### 3.5. Coproculture

As shown in Tables 5, 6, four sheep GIN genera were identified in varying proportions on the two farms examined: *Trichostrongylus*, *Teladorsagia*, *Haemonchus*, and *Chabertia*. In total, their representation before treatment was 43.67, 39, 11, and 6.33% in Farm 1 and 44.33, 43.67, 10, and 2% in Farm 2. These percentages did not change significantly after treatment in any of the tested groups (*p* > 0.05).

**Table 5:** Percentage of sheep nematode third-stage larvae (L_3_) for each treatment group at D0, D7, and D14–Farm 1.

Group	Day	*Trichostrongylus* (%)	*Teladorsagia* (%)	*Haemonchu*s (%)	*Chabertia* (%)
*M. piperita* EO	D0	56	30	8	6
	D7	51	33	12	4
	D14	43	41	5	11
Albendazole (control +)	D0	39	42	13	6
	D7	-	-	-	-
	D14	-	-	-	-
Sunflower oil (control +)	D0	36	45	12	7
	D7	34	47	9	10
	D14	31	48	10	11

No statistically significant differences were found when comparing values in different time points within groups, nor when comparing values for different groups on the same day in any of the groups tested (*p* > 0.05); EO, essential oil.

**Table 6:** Percentage of sheep nematode third-stage larvae (L_3_) for each treatment group at D0, D7, and D14–Farm 2.

Group	Day	*Trichostrongylus* (%)	*Teladorsagia* (%)	*Haemonchu*s (%)	*Chabertia* (%)
*M. piperita* EO	D0	45	47	6	2
	D7	39	49	8	4
	D14	48	35	11	6
Albendazole (control +)	D0	44	43	11	2
	D7	-	-	-	-
	D14	-	-	-	-
Sunflower oil (control +)	D0	44	41	13	2
	D7	40	45	11	4
	D14	42	47	8	3

No statistically significant differences were found when comparing values in different time points within groups, nor when comparing values for different groups on the same day in any of the groups tested (*p* > 0.05); EO, essential oil.

## 4. Discussion

The urgent problem associated with the development and spread of AR in sheep GINs requires actively searching for alternative strategies, whereby botanical anthelmintics are considered one of the most promising options (50). However, finding the most suitable plant formulation among the wide number of species, both from an efficacy and safety point of view, as well as from the aspect of price, is a challenging task. Thus, EOs and extracts from a wide number of plants have been tested so far on the efficacy against sheep GINs, as discussed earlier (32, 33). As can be noticed, most of these studies refer to *in vitro* testing. Although these tests are appropriate for the initial evaluation of anthelmintic potential due to their speed, reproducibility, easy application, absence of animals, and price (48), *in vivo* tests and evaluation of safety are also crucial parts of the process of developing a new anthelmintic drug and are needed before the formulation is introduced into practice (51). The choice of assays is also important, with the EHT and FECRT being recommended due to their accuracy and reliability. In fact, these tests are most commonly used for such studies (23, 52).

Considering the number of studies conducted so far and their results, the EO of *M. piperita* can be considered one of the potential candidates for the development of a new anthelmintic agent. Thus, in a study by Katiki et al. (37), it showed significant activity against *Haemonchus contortus* on the EHT with an obtained IC_50_ value of 0.26 mg/mL. Along with the ovicidal activity, EO also exhibited an effect on larval development and feeding with IC_50_ values of 0.26 and 0.07 mg/mL, respectively. Next, a study by Chagas et al. (38) has demonstrated moderate ovicidal (IC_50_ = 1.44 mg/mL) but high larvicidal (IC_50_ = 0.10 mg/mL) activity of the aforementioned EO, also against *H. contortus*. In our last study (39), *M. piperita* EO showed a strong, dose-dependent (*R*^2^ = 0.98) activity against a mixture of sheep GIN genera (*Hemonchus*, *Trichostrongylus*, *Teladorsagia,* and *Chabertia*) with an inhibition of egg hatchability varying from 72.5–99.8% (IC_50_ = 0.28 mg/mL) in the concentration range of 0.049–50 mg/mL. From that perspective, the results of the EHT in the present study showed a lower ovicidal effect of *M. piperita* EO, which may be attributed to the use of different EO samples in comparison with that study. In addition, in the present study, we used a different method to obtain concentrations (dilution method in comparison with micropipettes in the last study).

In the FECRT, the tested formulation (EO diluted in sunflower oil) exhibited an anthelmintic activity of 46.04% in total on Day 14 at the tested dose of 150 mg/kg (Table 3). As in our study performed with oregano EO (41), the efficacy was better in Farm 2 compared with Farm 1 (63.35 and 28.73%, respectively, on D14) (Graph 3), which can be explained by the differences in animal husbandry. That is, on the second farm, sheep were kept in boxes during the treatment, which facilitated the manipulation of animals and the application of formulations. In contrast, in the first farm, sheep were completely kept free-range. Other factors such as differences in feed (due to differences in animal husbandry), and consequently composition and volume of rumen content, may also affect the results. On the other hand, albendazole showed a high effect (>95%) on both farms on Days 7 and 14, indicating that anthelmintic resistance did not develop in the tested farms. The lower efficacy of the tested sample compared with the positive control can be explained by interactions of phytochemicals along the gastrointestinal tract and thus their partial inactivation and consecutive limited bioavailability (29). Next, the high standard deviation values of EPGs in both farms in all treatment groups suggest a large variation in the degree of parasite burden of individual animals. This is usually a characteristic of GINs, where the largest number of parasites accumulates in a smaller number of animals in certain herd (3). On the other hand, this suggests the importance of TST strategies during the treatments (19–21).

According to the GC–MS analyzes, menthol and menthone were most responsible for the anthelmintic effect shown, as their percentage representations were 32.6 and 22.0%, respectively. Indeed, these two compounds are considered the most important for various pharmacological properties of *M. piperita* EO and its wide use (35). However, pure menthol failed to reduce the EPG of *H. contortus* during *in vivo* testing. In contrast, the whole EO of *M. arvensis*, whose main component was menthol (86.7%), showed an efficacy of approximately 50% on Days 1, 14, and 21 (38). In other similar studies, whole EOs were also usually more effective than their isolated major components, as shown for *Croton Zehtneri* - anethole (53) and *Thymus vulgaris*–thymol (54). This suggests the importance of all EO compounds for their activity, potentially due to their synergistic effect. The absence of an effect of pure menthol in mentioned research was explained by its limited bioavailability and by the fact that menthol is excreted in the form of glucuronides via urine (38).

In comparison with the composition of *M. piperita* EO in our previous study (39), differences in the present compounds and their percentages may be observed. That is, the main compounds of that sample were piperitone (25.4%) and trans-dihidrocarvone (14.6%). In other mentioned studies, the chemical composition of *M. piperita* EO corresponds more to the sample of the present study. Thus, menthol (42.5%) followed by menthone (27.4%) were the main compounds of the sample in a study by Katiki et al. (37), and menthol (30.5%) was also the main compound of the sample in a study by Chagas et al. (38). This suggests that the composition of EO may vary considerably, even among those obtained from the same plant species. From that aspect, various factors such as the age and part of the plant, geographical origin, precipitation, light, and soil properties that refer to pH, structure, and salinity, as well as the presence of various organisms and microorganisms in a plant environment may be involved. In addition, the methods used for the extraction of oils, the producers from whom the oils are sourced, and the way they were stored prior to their use may also affect chemical composition (55, 56). Consequently, these factors may also lead to smaller or larger differences in efficacies, as can be noticed if the results of the mentioned studies, including the present one, are compared. The question that arises is to what extent these variations may hinder the development of commercial preparation, and whether ways to standardize the composition of certain EOs should be a topic of further studies.

Despite their increasing popularity, one of the main problems associated with the wider use of EOs in practice appears to be the lack of toxicity studies (33). Potential toxic and side effects of EOs and their constituents are usually tested in experimental animals such as rodents, whereas acute toxicity tests conducted so far in rats showed that most EOs have an LD_50_ of 1–20 g/kg, indicating their low toxicity (57). However, studies evaluating the effects on the host are rarely conducted. In the present study, the results of the preliminary toxicity tests showed the absence of side and toxic effects of *M. piperita* EO on sheep, i.e., their behavior, hematological parameters, as well as kidney and liver functions. This suggests the safety of the use of the applied formulation in sheep, at least when it comes to short-term effects. The same results were obtained for EO of lemongrass (*Cymbopogon schoenanthus* L.) applied to sheep (180 and 360 mg/kg, p.o.), and for the encapsulated combination of anethole and carvone applied to lambs (20 and 50 mg/kg, p.o.), with the absence of toxic effects on animal behavior, as well as liver and kidney functions, suggesting their safety (58, 59).

In a study by Chagas et al. (38), no toxicity symptoms were observed in sheep treated with *M. arvensis* EO. This and the results of the present study may be considered to be expected since the main compound of both of these EOs, menthol, has very low acute oral toxicity (LD_50_ > 2000 mg/kg b.w.) and is therefore generally recognized as safe by the FDA (U.S. Food and Drug Administration). Moreover, the FDA has approved the use of menthol in food (38, 60). According to the Assessment Report on *Mentha x piperita* L., *folium* and *aetheroleum* (61) can be safely used in humans at the recommended doses (oral, cutaneous, and inhalation) for the treatment of various gastrointestinal disorders or the symptomatic relief of mild tension-type headaches. However, although *Mentha* species are considered safe, special attention should be paid to the content of pulegone and menthofuran in the formulation due to their reported hepato-toxicity (62, 63). Thus, according to the Public Statement on the use of herbal medicinal products containing pulegone and menthofuran (64), the intake of their combination in 37.5 mg/person/day in humans is considered acceptable for herbal-medicinal products, which is set up as a limit for a life-long exposure. Such studies should be provided for animals including sheep.

The results of the coproculture examination showed the presence of four GIN genera before the treatment on the tested farms: *Trichostrongylus* (44%), *Teladorsagia* (41.33%), *Haemonchus* (10.5%), and *Chabertia* (4.17%). Since their percentage representation did not change significantly (*p* > 0.05) after the treatment, it can be concluded that the treatment with *M. piperita* EO is not specific for only a single genus. However, the results of some other studies showed higher activity of EOs against *H. contortus* in comparison with *Trichostrongylus* spp. after per-oral administration of the formulations (65–67), suggesting that there may be differences in sensitivity on EOs within the GIN genera (e.g., due to their position in the gastrointestinal tract). Anyhow, the mechanism of nematicidal activity of *M. piperita* EO compounds is still not fully elucidated. In a study by Choudhary et al. (68), menthol was shown to potentiate acetylcholine and levamisole responses in the receptor sensitive to levamisol but not to nicotine. The same study also showed that menthol can significantly potentiate the contraction of *Ascaris suum* somatic muscle strips at each concentration of acetylcholine. A study by Khan et al. (69) showed that methanol and the extract of *M. arvensis* cause high apoptotic effect in the muscles, gonads, and uterus (eggs) of the free-living nematode *Caenorhabditis elegans*, which was used as a model organism. The results also showed that stress genes (gst-4 and hsp-16.2) were highly expressed in the affected nematodes compared to normal *C. elegans*.

Although the applied oil significantly reduced the number of EPG after the treatment, with the achieved *in vivo* efficacy being one of the highest obtained for EOs against GINs (32, 33), it is still insufficient for its independent use. However, studies conducted so far showed that none of the aforementioned possible control methods appears to be sustainable when applied alone (6). Thus, as discussed earlier, it is generally agreed that a combination of different strategies in the form of integrated parasite management is the only sustainable solution for long-term parasite control (6, 16–19, 70). An example is the results obtained in a mentioned study by Choudhary et al. (68), where the positive allosteric modulatory properties of menthol were proven, which could be used in combination therapy with cholinergic anthelmintics.

From all the above, results obtained in the present study suggest that *M. piperita* EO can play a valuable role in an integrated approach to control parasites. In general, the use of botanical anthelmintics offers many advantages, such as their rich chemical composition with a lot of compounds belonging to different chemical groups (lower susceptibility to the development of resistance), their natural origin (lower amount of residues in animal products and the environment), suitable price, etc. (13, 14, 48, 50). Moreover, *in vivo* efficacy of *M. piperita* EO could be further improved with the use of an encapsulation technique that can protect its active ingredients from degradation, and thus enable their higher bioavailability (71, 72). On the other hand, better results can also be achieved by increasing the dose or using multiple applications over several consecutive days instead of single administration although the use of a higher dose or multiple administration should also be evaluated from the toxic point of view. Efficacy can also be improved, as well as control release enabled by other ways of applications such as lick blocks that contain plant-based compounds, and may provide long-term use (73).

## 5. Conclusion

The development and spread of anthelmintic resistance in nematodes require an urgent search for alternatives. The results of the present study suggest that *M. piperita* EO is suitable for use against GINs in sheep based on the efficacy and preliminary toxicity tests performed. Therefore, in combination with other strategies (rational use of anthelmintics or other alternatives), the tested formulation could play a valuable role in an integrated, environmentally friendly control of these parasites. Further studies should be performed to confirm the safety after long-term use of peppermint oil in practice to achieve even higher efficacy in field trials, as well as to test its efficacy against GINs resistant to commercial drugs.

## Data availability statement

The original contributions presented in the study are included in the article/Supplementary material, further inquiries can be directed to the corresponding authors.

## Ethics statement

The study was conducted according to the guidelines of the Declaration of Helsinki and approved by the Ethics Committee of the University of Naples (PG/2021/0130480, 16 December 2021). The studies were conducted in accordance with the local legislation and institutional requirements. Written informed consent was obtained from the owners for the participation of their animals in this study.

## Author contributions

FŠ: conceptualization, methodology, formal analysis, investigation, data curation, and writing original draft preparation. SK: conceptualization, resources, and writing review and editing. DS: conceptualization, validation, and supervision. RR: conceptualization, methodology, and resources. NS: investigation, resources, and writing review and editing. DO: investigation and resources. LR: methodology, validation, writing review and editing, and supervision. EC: methodology and investigation. MM: methodology and validation. GC: validation and supervision. AB: conceptualization, methodology, investigation, data curation, and writing review and editing. All authors contributed to the article and approved the submitted version.

## Funding

This research was cofounded by the Ministry of Education, Science, and Technological Development of the Republic of Serbia, rescript No. 451–03-1183/2021–14.

## Conflict of interest

The authors declare that the research was conducted in the absence of any commercial or financial relationships that could be construed as a potential conflict of interest.

## Publisher’s note

All claims expressed in this article are solely those of the authors and do not necessarily represent those of their affiliated organizations, or those of the publisher, the editors and the reviewers. Any product that may be evaluated in this article, or claim that may be made by its manufacturer, is not guaranteed or endorsed by the publisher.
