# Supplementary material for: In vitro and in vivo anthelmintic efficacy of peppermint (Mentha x piperita L.) essential oil against gastrointestinal nematodes of sheep
Source: Front Vet Sci. 2023 Aug 10;10:1232570. doi: 10.3389/fvets.2023.1232570 (PMC10472939; doi:10.3389/fvets.2023.1232570)
Supplement: Supplementary file 1 [file Table_1.pdf]

TABLE S1 The effect of *Mentha x piperita* (L.) essential oil on hematological parameters in treated animals–in total from both examined farms.

Parameters	Reference values	Day	*Menta x piperita* (L.) EO	Albendazole, control (+)	Sunflower oil, control (-)
WBC (K/uL)	4.0 – 12.0	0.	8.04 ± 1.8	9.59 ± 1.6	8.78 ± 2.9
		14.	8.08 ± 2.3	9.45 ± 2.6	8.74 ± 2.3
RBC (M/uL)	8.0 – 16.0	0.	**7.78 ± 0.9**	**7.50 ± 0.5**	8.01 ± 1.0
		14.	**7.63 ± 0.6**	**7.66 ± 0.6**	**7.45 ± 1.1**
Hgb (g/dL)	8.0 – 16.0	0.	10.6 ± 1.3	10.4 ± 0.5	11.7 ± 1.8
		14.	10.9 ± 0.8	11.1 ± 0.8	11.0 ± 1.7
Hct (%)	24.0 – 50.0	0.	26.5 ± 2.9	26.4 ± 1.6	29.4 ± 4.7
		14.	26.1 ± 2.0	27.3 ± 1.8	27.4 ± 4.2
MCV (fL)	23.0 – 48.0	0.	34.1 ± 1.4	35.2 ± 1.7	36.8 ± 2.4
		14.	34.3 ± 1.8	35.7 ± 2.0	36.8 ± 1.8
MCH (Pg)	9.0 – 12.0	0.	**13.6 ± 0.4**	**13.9 ± 0.6**	**14.6 ± 0.8**
		14.	**14.3 ± 0.5**	**14.5 ± 0.7**	**14.7 ± 0.8**
MCHC (g/dL)	31.0 – 38.0	0.	**39.9 ± 1.2**	**39.5 ± 1.0**	**39.7 ± 0.8**
		14.	**41.8 ± 1.4**	**40.5 ± 0.7**	**40.1 ± 0.9**
RDW (%)	-	0.	15.5 ± 0.8	15.5 ± 0.5	15.9 ± 0.7
		14.	15.4 ± 0.7	15.8 ± 0.5	15.7 ± 0.8
Plt (K/uL)	250 - 750	0.	656.9 ± 220.9	517.0 ± 119.0	545.1 ± 189.1
		14.	594.6 ± 183.1	468.2 ± 142.0	483.0 ± 207.7
MPV (fL)	-	0.	12.2 ± 1.4	11.3 ± 1.5	11.1 ± 1.3
		14.	12.1 ± 1.5	11.4 ± 1.6	10.8 ± 1.3
Pct (%)	-	0.	0.70 ± 0.2	0.60 ± 0.6	0.58 ± 0.2
		14.	0.66 ± 0.2	0.54 ± 0.2	0.46 ± 0.1
PDW (%)	-	0.	3.04 ± 4.7	5.43 ± 4.5	5.83 ± 8.0
		14.	4.47 ± 4.9	4.59 ± 5.0	9.23 ± 9.6
Lin (K/uL)	-	0.	4.39 ± 1.5	4.94 ± 1.3	4.16 ± 1.1
		14.	4.78 ± 1.4	5.41 ± 2.5	4.73 ± 1.6
Lin (%)	40 - 75%	0.	53.8 ± 11.2	51.8 ± 12.0	52.8 ± 12.3
		14.	58.8 ± 6.2	56.4 ± 17.1	54.2 ± 6.8
Gra (K/uL)	-	0.	3.65 ± 1.0	4.65 ± 1.4	4.09 ± 2.4
		14.	3.31 ± 1.1	4.04 ± 1.7	4.01 ± 1.2
Gra (%)	-	0.	46.3 ± 1.1	48.2 ± 12.0	47.2 ± 12.3
		14.	41.2 ± 6.2	43.6 ± 17.1	45.8 ± 6.8

No statistically significant differences were found when comparing values at Day 14 and Day 0 within groups, nor when comparing values between different treatment groups on the same time point ($p>0.05$); EO - essential oil; WBC - white blood cells; RBC – red blood cells; Hgb – haemoglobin; Hct – haematocrit; MCV – mean corpuscular

volume; MCH – mean corpuscular haemoglobin; MCHC – mean corpuscular haemoglobin concentration; RDW – red cell distribution width; Plt – platelet count; MPV – mean platelet volume; Pct – procalcitonin; PDW – platelet distribution width; Lin – lymphocytes; Gra – granulocytes.
